# ADN-1184, a monoaminergic ligand with 5-HT_6/7_ receptor antagonist action, exhibits activity in animal models of anxiety

**DOI:** 10.1007/s00210-016-1229-3

**Published:** 2016-03-16

**Authors:** Anna Partyka, Anna Wasik, Magdalena Jastrzębska-Więsek, Paweł Mierzejewski, Przemysław Bieńkowski, Marcin Kołaczkowski, Anna Wesołowska

**Affiliations:** Department of Clinical Pharmacy, Jagiellonian University Medical College, 9 Medyczna Street, 30-688 Cracow, Poland; Department of Pharmacology, Institute of Psychiatry and Neurology, 9 Sobieskiego Street, 02-957 Warsaw, Poland; Department of Pharmaceutical Chemistry, Jagiellonian University Medical College, 9 Medyczna Street, 30-688 Cracow, Poland; Adamed Ltd, Pieńków 149, 05-152 Czosnów, Poland

**Keywords:** BPSD, Anxiety, Depression, Antipsychotic

## Abstract

Behavioral and psychological symptoms of dementia (BPSD) include apathy, sleep problems, irritability, wandering, elation, agitation/aggression, and mood disorders such as depression and/or anxiety. Elderly patients are usually treated with second-generation antipsychotics; however, they present not enough efficacy against all symptoms observed. Hence, there still is an unmet need for novel pharmacotherapeutic agents targeted BPSD. A novel arylsulfonamide derivative ADN-1184 has been developed that possesses a preclinical profile of activity corresponding to criteria required for treatment of both psychosis and depressive symptoms of BPSD without exacerbating cognitive impairment or inducing motor disturbances. To broaden its pharmacological efficacy toward anxiety symptoms, its anxiolytic properties have been examined in common animal preclinical models in rats and mice. ADN-1184 significantly increased the number of entries into open arms measured in the elevated plus-maze test; however, it simultaneously increased parameters of exploratory activity. In the Vogel conflict drinking test, ADN-1184 dose-dependently and significantly increased the number of shocks accepted and the number of licks. Moreover, in mice, it also had specific anxiolytic-like activity in the four-plate test, and only negligible one at a specific mid-range dose measured in the spontaneous marble burying test. The obtained findings reveal that ADN-1184 displays anxiolytic-like activity in animal models of anxiety which employed punished stimuli. In its unusual combination of some anxiolytic action with already proven antipsychotic and antidepressant properties, and lack of any disruptive impact on learning and memory processes and motor coordination, ADN-1184 displays a profile that would be desired for a novel therapeutic for BPSD.

## Introduction

Dementia is a clinical syndrome that expresses itself in three areas, i.e., cognitive deficits, psychological and behavioral disturbances, and difficulties in carrying out daily functions (De Deyn and Wirshing 2001). Behavioral and psychological symptoms of dementia (BPSD) include apathy, sleep problems, irritability, wandering, elation, agitation/aggression, and mood disorders such as depression and/or anxiety. At least one symptom of BPSD is reported in about 80 % patients with dementia (Proitsi et al. 2011). The prevalence of anxiety is rated as 38 % in Alzheimer disease and up to 72 % in vascular dementia (Ballard et al. 2000).

BPSD affect the quality of life of older people and their caregivers. Pharmacological treatment ought to be considered when first-line non-pharmacological management fails or when there is a significant risk (e.g., physical aggression, agitation, or psychosis) (Cheung and Stapelberg 2011). Elderly patients are usually treated with second-generation antipsychotics (SGAs) that produce fewer extrapyramidal side-effects and have better efficacy against negative symptoms than classical neuroleptics (Byerly et al. 2001; Liperoti et al. 2008; Kołaczkowski et al. 2014b). Despite the global and embedded use of antipsychotics for BPSD treatment, the evidence and rationale for the use of this drug class is proven to be modest.

A recent meta-analysis based on 14 placebo-controlled trials of elderly patients with BPSD revealed only modest positive effects for risperidone, olanzapine, and aripiprazole, all SGAs (Maher et al. 2011). Moreover, SGAs may produce adverse effects including cardiovascular and metabolic ones (Nobili et al. 2009; Schulze et al. 2013). These drugs may also worsen cognitive function, especially in the case of elderly patients who already suffer from memory deficits (Jeste et al. 2008; Vigen et al. 2011). Some SGAs might be an option for the treatment of depressive symptoms (Moller 2000, 2005a, b; Leucht et al. 2009); however, they were not developed to provide alleviation of mood deficits in elderly patients. There still is an unmet need for novel pharmacotherapeutic agents targeted for treatment of BPS—an increasingly challenging problem connected with the aging populations in many developed countries.

In Kołaczkowski et al. (2014a), we described a novel arylsulfonamide derivative (ADN-1184) that possesses a preclinical profile of activity corresponding to criteria required for treatment of both psychosis and depressive symptoms of BPSD without exacerbating cognitive impairment or inducing motor disturbances. As no animal model of BPSD is available, ADN-1184’s potential antipsychotic properties have been evaluated in a range of procedures, including hyperactivity and stereotypies induced by MK-801, procedures relevant to dementia patients who suffer from psychoses of glutamatergic origin (Bardin et al. 2007; Siegel 1978) as well as conditioned avoidance response (Kołaczkowski et al. 2014a). In all these models, ADN-1184 demonstrated an antipsychotic-like potency similar to or even stronger than that produced by SGAs and tested in the same experimental conditions (Kołaczkowski et al. 2014a). Moreover, ADN-1184 was also active in a classic model of potential antidepressant activity—the forced swim test—showing potency of the same order of magnitude as that of imipramine. Furthermore, its active antidepressant doses were lower than those in models of antipsychotic-like activity. Simultaneously, ADN-1184 did not impair the passive avoidance response in contrast to SGAs, which elicited dose-dependent disruption in that model. While being active in models of psychosis, mood deficit and memory performance, ADN-1184 additionally did not elicit catalepsy or a decrease in locomotor activity when administered at antipsychotic and antidepressant doses. ADN-1184 only began to decrease locomotor action at a dose of 30 mg/kg and, at the same dose, weakly elicited catalepsy (Kołaczkowski et al. 2014a).

ADN-1184 in vivo profile, combining antipsychotic- and antidepressant-like activities without cognitive or motor impairments, appears to stem from its unusual receptor combination resulting from potent blockade of 5-HT_6_, 5-HT_7_, and 5-HT_2A_ receptors, and modest antagonism of dopamine D_2_ and D_3_ receptors in the absence of anticholinergic effects. ADN-1184 also antagonizes α_1_-adrenergic receptors which may contribute to its antipsychotic efficacy, widens the therapeutic window with regard to extrapyramidal side-effects (Wadenberg et al. 2000), and helps to reduce agitation and aggressive behavior (Wang et al. 2009) seen in elderly patients with BPSD. Antagonism of peripheral α_1_-adrenergic receptors is associated with adverse cardiovascular effects, especially in elderly people; thus, this needs to be taken into consideration in the treatment of patients with potentially fragile cardiac function.

The above observations suggest that the combined multireceptor functional profile of ADN-1184 and its potential antipsychotic/antidepressant action observed in rats might offer an effective and broad-based platform for the control of mood deficit states. Inasmuch as anxiety is a common and comorbid symptom of depression, the present work discusses the potential anxiolytic-like activity of ADN-1184 detected in a wide range of rodent preclinical models of anxiety. Its activity has been compared to action of three SGAs (risperidone, olanzapine, and aripiprazole) for which small but statistically significant benefits have been observed in a recent meta-analysis (Maher et al. 2011) for the treatment of behavioral symptoms associated with dementia in elderly patients.

## Materials and methods

### Animals

The experiments were conducted on male Wistar rats (230–300 g) purchased from Charles River Laboratories (Germany) and male Albino Swiss mice (21–25 g) obtained from a licensed breeder (Staniszewska, Poland). Animals were kept in a group of four (rats) or 10 (mice) in standard plastic cages and housed in a room under controlled conditions (temperature 21 ± 2 °C, humidity 50–60 %, under a 12-h light/dark cycle, lights on at 8.00 a.m.). Standard lab chow (Labofeed H, WPiK, Kcynia, Poland) and tap water were available ad libitum unless otherwise stated. All investigations were performed between 9:00 a.m. and 4:00 p.m. in soundproof experimental rooms under dim light and continuous white noise (65 dB). Behavioral parameters were recorded automatically by microcomputers or manually scored by observers unaware of the treatment applied. Tested animals were used only once. Treatment of animals in the present studies was in full accordance with the ethical standards laid down in respective Polish and European (Directive no. 86/609/EEC) regulations. All procedures were approved by a local ethics committee.

### Drugs

ADN-1184 (hydrochloride salt), risperidone, olanzapine, and aripiprazole provided by Adamed Ltd. (Pieńków, Poland), were suspended in a 1 % Tween 80 (Sigma-Aldrich) and administered intraperitoneally (i.p.) in a volume of 2 ml/kg (rats) or 10 ml/kg (mice). The drugs were injected 60 min (rats) or 30 min (mice) before the experiment. Control animals received vehicle according to the same procedure. All solutions were prepared immediately prior to use and protected from the light.

### Behavioral procedures in rats

#### Elevated plus-maze (EPM) test

The testing procedure was based on a method described by Pellow and File (1986). EPM test is an “unconditional” anxiety-like test based on rodents’ natural aversion to heights and open spaces. Plus-maze apparatus (an automated device produced by Campden Instruments Ltd. (UK) made of durable, high density, non-porous black plastic, elevated to a height of 50 cm, consisted of two open arms (50 × 10 cm) and two closed arms (50 × 10 cm, and 30-cm high walls), arranged so that the two arms of each type were opposite each other. Floor of the plus-maze was made of infrared transparent material what means that there are no visible sensors. Plus-maze apparatus was connected to PC software by control chassis. The experiments were conducted under dim light (only the center of the maze was illuminated with low-intensity light 30 lux measured on the maze level) and continuous white noise (65 dB). Each rat was gently placed in the center of the plus-maze, facing one of the closed arms, immediately after a 5-min adaptation period in a plastic black box (60 × 60 × 35 cm), to increase the overall activity in the EPM. During a 5-min test period, automated Motor Monitor System recorded the number of entries into the closed and open arms and the time spent in either type of the arms. The device counted an effective arm-entry when the four paws of a rat were into any arm. The maze was thoroughly cleaned after each trial.

#### Exploratory activity measured in the EPM test

To assess an influence of a tested compound on general exploratory activity of rats and to control possible changes within, total ambulation (the total distance covered by a rat, and ambulation along X and Y axis) was taken during a 5-min test period (i.e., the time equal to the observation period in the EPM test). The experiment was performed using EPM apparatus (details see above).

#### Vogel conflict drinking test

The testing procedure based on a method described by Vogel et al. (1971) was performed using Anxiety Monitoring System “Vogel test” produced by TSE Systems. Apparatus was consisted of a polycarbonate cage (dimensions 26.5 _15 _ 42 cm), equipped with a grid floor made from stainless steel bars and a drinking bottle containing tap water. Experimental chambers (two) were connected to PC software by control chassis and a device that generates electric shocks. In this “conditional” model, an electric shock as noxious stimulus is applied. The testing procedure consisted of 2-day habituation/adaptation and an exact test. On the first day of the experiment, the rats were adapted to the test chamber for 10-min adaptation period during which they had free access to the drinking bottle followed by a 24-h water deprivation period. Afterwards, they were allowed a 30-min free-drinking session in their home cages. This protocol of 24-h deprivation and adaptation period was repeated on the second day. On the third day, animals were place again in the test chamber 60 min after administration of vehicle/ADN-1184 and were given free access to drinking bottle during 5 min. Recording the data started immediately after the first lick, and every 20 licks rats were punished with an electric shock (0.5 mA, lasting 1 s). The impulses were released via the spout of the drinking bottle. The number of licks and the number of shocks received throughout a 5-min experimental session were recorded automatically.

#### Hot plate and free-drinking tests

To exclude possible drug-induced changes in shock sensitivity or an increasing influence on thirst drive which can lead to false positive results in the Vogel conflict drinking test, stimulus threshold and water consumption during a free-drinking session were determined in separate groups of rats. In either of those two studies, the rats were manipulated similarly to the Vogel conflict drinking test, including two 24-h water deprivation periods separated by 10-min adaptation session in experimental cages and 30-min of water availability in their home cages. In the free-drinking test, each animal was allowed to freely drink from the drinking bottle and the amount of water (g) consumed during 5 min was recorded for each rat. The pain threshold was evaluated using hot plate test (Commat Ltd, Turkey) in rats. The plate was enclosed with a transparent Plexiglass cylinder (35-cm high) to keep the animal on the heated surface of the plate. The latency to pain reaction (lick a hind paw or jumping) when the rat was placed on a hot plate (52.5 ± 0.5 °C, 19-cm diameter) was measured. The rat was removed from the plate immediately upon visible pain reaction or if no response occurred within 30 s.

### Behavioral procedures in mice

#### Four-plate test (4-PT)

The four-plate apparatus (BIOSEB) consists of a cage (25 × 18 × 16 cm) floored by four identical rectangular metal plates (8 × 11 cm) separated from one another by a gap of 4 mm. The top of the cage is covered by a transparent Perspex lid that prevents escape behavior. The plates are connected to a device that can generate electric shocks. Following a 15-s habituation period, the animal’s motivation to explore a novel environment is suppressed by an electric foot shock (0.8 mA, 0.5 s) every time it moves from one plate to another during a 1-min test session. This action is referred to as a “punished crossing” and is followed by a 3-s shock interval, during which the animal can move across plates without receiving a shock (Aron et al. 1971).

#### Marble burying test (MBT)

The test was performed based on a method described by Broekkamp et al. (1986) with minor modifications. Mice were placed individually into plastic cages which were identical to their home cage containing a 5-cm layer of sawdust and 20 marbles (1.4-cm diameter) evenly spaced against the wall. After 30-min experimental period, mice were removed from cages and the number of marbles at least 2/3 buried was counted. The experiment was conducted on Albino Swiss mice.

### Data analysis

All the data are presented as the mean ± SEM. The statistical significance of the results was evaluated by a one-way analysis of variance (ANOVA), followed by Bonferroni’s comparison test (statistical significance set at *p* < 0.05). The data from the hot plate and free-drinking test was analyzed using a *t* test. The Statistica 10.0 software package for Windows (StatSoft, Tulsa, OK, USA) was used to analyze all data.

## Results

### EPM test

ADN-1184 at a dose of 0.3 mg/kg only, increased significantly the number of entries into the open arms. Risperidone at doses of 0.03 and 0.1 mg/kg statistically increased the percentage of entries into open arms. Olanzapine and aripiprazole produced no significant effects in that test; however, a tendency to increase the number of entries into open arms and the percentage of entries into the open arms, respectively, was observed (Table 1).

**Table 1:** Anxiolytic-like effects of the tested compounds in the EPM test in rats

Treatment	Dose (mg/kg)	Time (s) spent in the open arms	Percentage of time spent in the open arms	Number of entries into the open arms	Percentage of entries into the open arms
Vehicle ADN-1184	0	49.2 ± 7.4	25.7 ± 3.5	10.6 ± 2.0	31.6 ± 5.1
	0.1	68.8 ± 6.3	34.0 ± 5.4	13.1 ± 1.4	32.5 ± 4.4
	0.3	87.8 ± 11.4	44.0 ± 4.8	18.2 ± 2.2*	37.8 ± 2.8
	1	78.3 ± 2.4	36.0 ± 5.6	15.3 ± 1.6	37.8 ± 3.9
	3	73.5 ± 15.1	35.3 ± 2.9	11.0 ± 1.0	45.6 ± 4.6
		*F*(4,29) = 1.838, NS	*F*(4,29) = 2.122, NS	*F*(4,29) = 3.365, *p* < 0.05	*F*(4,29) = 1.866, NS
Vehicle Risperidone	0	43.1 ± 10.1	19.0 ± 4.9	8.5 ± 2.8	21.2 ± 4.5
	0.03	78.5 ± 11.3	33.6 ± 5.1	14.8 ± 2.1	38.0 ± 2.7*
	0.1	79.9 ± 8.9	33.1 ± 3.7	16.0 ± 1.7	37.3 ± 2.4*
	0.3	95.1 ± 19.4	40.4 ± 8.2	12.7 ± 2.1	36.4 ± 4.8
		*F*(3,25) = 2.515, NS	*F*(3,25) = 2.244, NS	*F*(3,25) = 2.145; NS	*F*(3,25) = 4.535, *p* < 0.05
Vehicle Olanzapine	0	84.3 ± 19.6	33.0 ± 7.5	13.0 ± 1.8	35.5 ± 5.3
	0.03	50.0 ± 12.8	19.8 ± 5.2	7.8 ± 1.1	29.1 ± 3.4
	0.1	94.8 ± 9.0	40.1 ± 4.4	16.3 ± 2.6	37.0 ± 6.0
	0.3	71.3 ± 12.3	27.8 ± 5.1	11.9 ± 2.4	40.5 ± 3.9
		*F*(3,25) = 2.118, NS	*F*(3,25) = 2.521, NS	*F*(3,25) = 3.070, *p* < 0.05	*F*(3,25) = 1.183, NS
Vehicle Aripiprazole	–	97.3 ± 20.6	40.4 ± 8.6	17.6 ± 4.0	39.0 ± 6.9
	0.3	123.8 ± 13.5	52.5 ± 4.7	18.8 ± 2.2	52.2 ± 4.5
	1	75.5 ± 14.7	32.6 ± 6.2	13.7 ± 1.9	36.4 ± 5.0
	3	89.5 ± 36.4	33.5 ± 12.3	9.5 ± 1.8	33.0 ± 4.1
		*F*(3,22) = 1.712, NS	*F*(3,22) = 1.556, NS	*F*(3,22) = 2.888, NS	*F*(3,22) = 3.141, *p* < 0.05

ADN-1184, risperidone, olanzapine, and aripiprazole were injected i.p. 60 min before the test. Values represent the mean ± SEM during the 5-min test session compared with vehicle group *p < 0.05 (ANOVA is followed by the Bonferroni’s post hoc test), *N* = 5–8

### Exploratory activity measured in the EPM test

Both parameters, i.e., total entries and total distance travelled presented in Table 2, measured simultaneously with anxiolytic-like activity using the automated version of the EPM, reflect general exploratory activity of rats. ADN-1184 at doses of 0.1, 0.3, and 1 mg/kg significantly increased one or two of parameters measured. The tested compound administered at a dose of 3 mg/kg had no influence on general exploratory activity. Similarly, all tested SGAs did not affect exploratory activity of rats measured in EPM (Table 2).

**Table 2:** Effects of the tested compounds on exploratory activity measured in the EPM in rats

Treatment	Dose (mg/kg)	Total entries	Total distance [cm]
Vehicle ADN-1184	0	29.0 ± 2.4	4613.0 ± 158.0
	0.1	40.6 ± 3.5	5686.6 ± 201.1*
	0.3	49.3 ± 4.5**	5944.5 ± 245.7**
	1	41.8 ± 3.9*	5519.0 ± 282.6
	3	25.9 ± 2.6	3777.9 ± 401.1
		*F*(4,29) = 7.853, *p* < 0.001	*F*(4,29) = 11.292, *p* < .0001
Vehicle Risperidone	0	35.5 ± 7.0	4465.3 ± 462.0
	0.03	39.9 ± 5.5	5268.0 ± 416.0
	0.1	42.9 ± 3.7	5072.5 ± 318.0
	0.3	35.1 ± 4.7	4524.7 ± 259.9
		*F*(3,25) = 0.516; NS	*F*(3,25) = 1.156; NS
Vehicle Olanzapine	0	38.2 ± 2.6	5078.7 ± 157.7
	0.03	26.6 ± 1.9	4552.5 ± 198.0
	0.1	43.4 ± 5.8	5401.1 ± 448.4
	0.3	30.6 ± 6.1	4591.6 ± 355.2
		*F*(3,25) = 2.680, NS	*F*(3,25) = 1.653, NS
Vehicle Aripiprazole	0	43.4 ± 6.7	5613.8 ± 222.9
	0.3	32.2 ± 6.5	4419.0 ± 467.4
	1	38.4 ± 2.5	4792.0 ± 132.9
	3	35.5 ± 2.8	4641.8 ± 260.9
		*F*(3,22) = 0.960, NS	*F*(3,22) = 2.651, NS

ADN-1184, risperidone, olanzapine and aripiprazole were injected i.p. 60 min before the test. Values represent the mean ± SEM during the 5-min test session compared with vehicle group *p < 0.05 (ANOVA is followed by the Bonferroni’s post hoc test), *N* = 6–8

### Vogel conflict drinking test

**Fig. 1:**
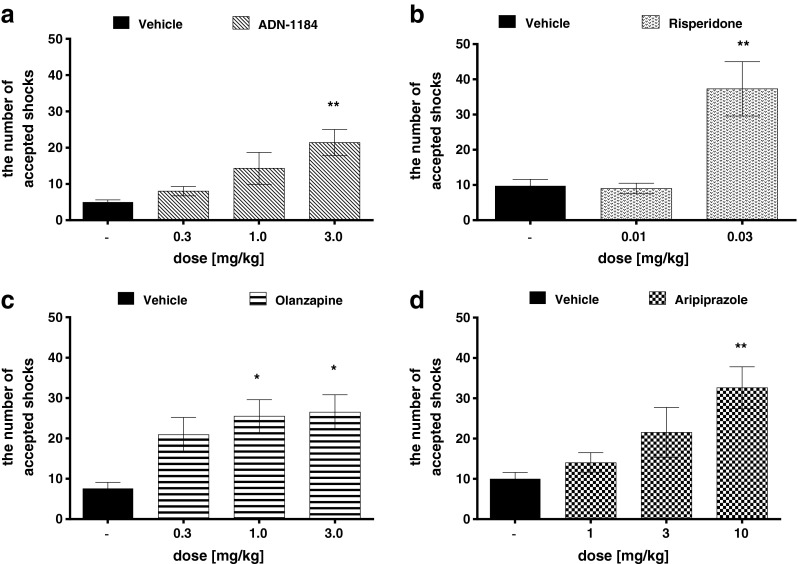
Anxiolytic-like effect of the tested compounds in the Vogel conflict drinking test in rats (the number of shocks accepted during 5 min). ADN-1184, risperidone, olanzapine, and aripiprazole were injected i.p. 60 min before the test. Values represent the mean ± SEM compared to the respective vehicle groups *p < 0.05; **p < 0.001 (ANOVA is followed by the Bonferroni’s post hoc test), *N* = 7–8

**Fig. 2:**
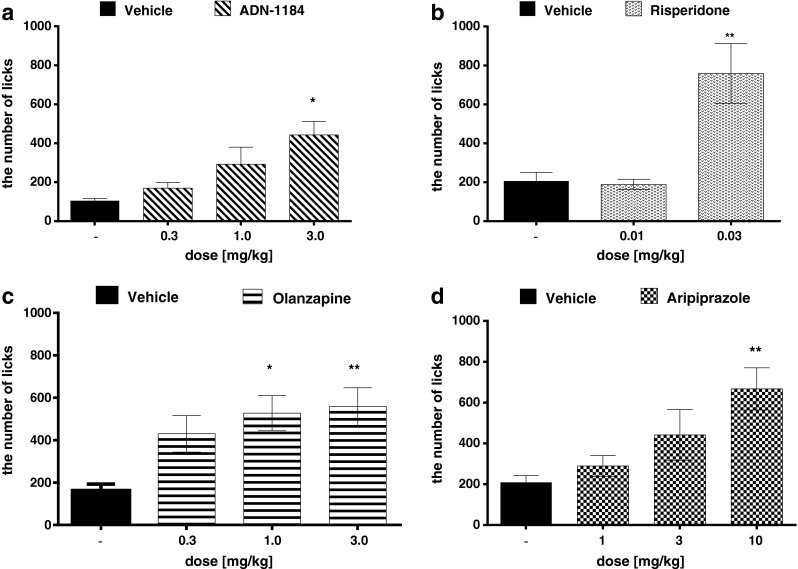
Anxiolytic-like effect of the tested compounds in the Vogel conflict drinking test in rats (the number of licks during 5 min). ADN-1184, risperidone, olanzapine, and aripiprazole were injected i.p. 60 min before the test. Values represent the mean ± SEM compared to the respective vehicle groups *p < 0.05; **p < 0.001 (ANOVA is followed by the Bonferroni’s post hoc test), *N* = 7–8

ADN-1184 at a dose of 3 mg/kg significantly increased the number of shocks accepted (ANOVA *F*(3,25) = 5.391, *p* < 0.01) and the number of licks (ANOVA *F*(3,25) = 5.761, *p* < 0.01) during 5-min experimental evaluation. Lower doses of ADN-1184, i.e., 0.3 and 1 mg/kg were ineffective in that test but the measured effect was dose-dependent (Figs. 1a and 2a). Risperidone at a dose of 0.03 mg/kg (ANOVA *F*(2,28) = 3.504, *p* < 0.01 and *F*(2,28) = 3.454, *p* < 0.01), olanzapine at doses of 1 and 3 mg/kg (ANOVA *F*(3,31) = 4.248, *p* < 0.05 and *F*(3,31) = 4.159, *p* < 0.05) and aripiprazole at a dose of 10 mg/kg (ANOVA *F*(3,23) = 6.079, *p* < 0.01 and *F*(3,23) = 5.922, *p* < 0.01) significantly increased the number of shocks accepted and the number of licks measured in the Vogel test, respectively (Figs. 1b–d and 2b–d).

### Hot-plate and free-drinking tests

ADN-1184, risperidone, olanzapine, and aripiprazole administered at doses effective in the Vogel conflict drinking test, i.e., 3, 0.03, 1, 3, and 10 mg/kg, respectively, had no effect on pain reaction time measured in the hot plate test as well as they did not change the amount of water consumed by deprived rats as compared with control animals, during 5-min session (Table 3).

**Table 3:** Effects of the tested compounds in the hot plate and water consumption tests in water-deprived rats

Treatment	Dose	Hot plate test	Water consumption
	(mg/kg)	Time of reaction (s)	(g/5 min))
Vehicle	0	8.1 ± 0.7	5.3 ± 0.3
ADN-1184	3	7.8 ± 0.9	5.6 ± 0.3
		^a^ *F*(1,12) = 0.844, NS	^a^ *F*(1,13) = 0.292, NS
Risperidone	0.03	7.1 ± 0.7	5.7 ± 0.9
		^a^ *F*(1,11) = 0.518, NS	^a^ *F*(1,12) = 1.006, NS
Olanzapine	1	8.4 ± 0.8	5.1 ± 0.4
	3	8.6 ± 0.6	5.6 ± 0.5
		^b^ *F*(2,20) = 1.480, NS	^b^ *F*(2,22) = 0.964, NS
Aripiprazole	10	7.5 ± 0.8	5.4 ± 0.9
		^b^ *F*(1,12) = 0.960, NS	^b^ *F*(1,12) = 1.051, NS

ADN-1184, risperidone, olanzapine and aripiprazole were injected i.p. 60 min before the test. Values represent the mean ± SEM of time reaction in the hot plate test and amount of water consumed during 5 min test session. The data were analyzed using ^a^ *t* test and ^b^one-way ANOVA followed by Bonferroni’s post hoc test, *N* = 7–8

### 4-PT

**Fig. 3:**
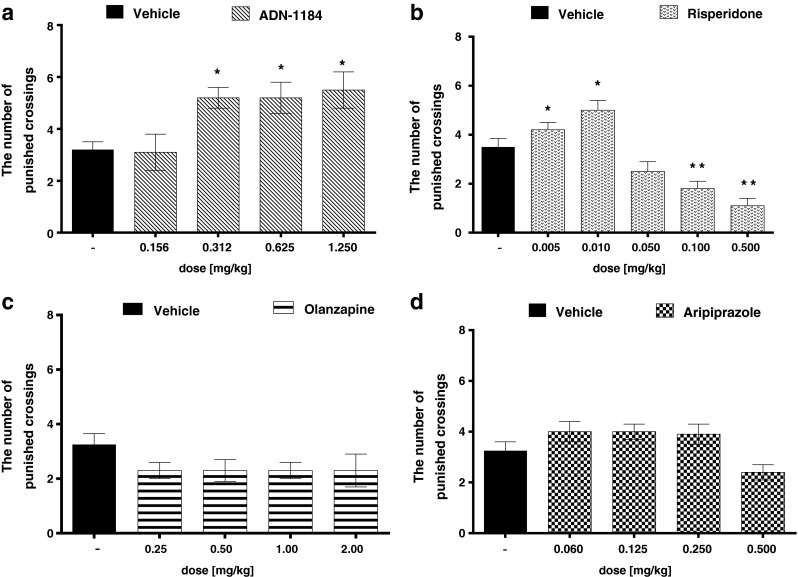
Anxiolytic-like effect of the tested compounds in the 4-PT in mice. ADN-1184, risperidone, olanzapine, and aripiprazole were injected i.p. 30 min before the test. Values represent the mean ± SEM compared to a vehicle group *p < 0.05; **p < 0.01 (ANOVA is followed by the Bonferroni’s post hoc test), *N* = 8–11

ADN-1184 given at doses of 0.312, 0.625, and 1.25 mg/kg, dose-dependently and significantly increased the number of punished crossings (ANOVA *F*(4,42) = 5.023, *p* < 0.01). There was no difference between control group and the dose 0.156 mg/kg of ADN-1184 (Fig. 3a). Risperidone at doses of 0.005 and 0.01 mg/kg produced anxiolytic-like activity measured in that test (ANOVA *F*(2,23) = 4.963, *p* < 0.05) while higher doses significantly decreased the number of punished crossings (ANOVA: *F*(3,36) = 9.013, *p* < 0.0001) (Fig. 3b). Olanzapine tested at doses of 0.25–2 mg/kg (ANOVA *F*(4,53) = 3.123, *p* < 0.05) and aripiprazole at doses of 0.06–0.5 mg/kg (ANOVA *F*(4,49) = 4.856, *p* < 0.01) produced no statistical anxiolytic-like effect in that test. In the case of aripiprazole, a tendency to increase the number of punished crossings could be observed; however, the results did not reach significant level (Fig. 3c, d).

### MBT

**Fig. 4:**
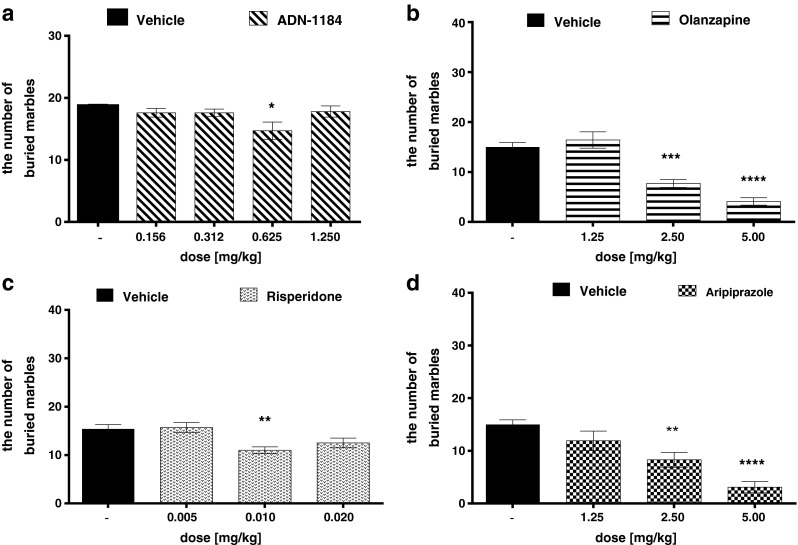
Anxiolytic-like effect of the tested compounds in the MBT in mice. ADN-1184, risperidone, olanzapine and aripiprazole were injected i.p. 30 min before the test. Values represent the mean ± SEM compared to a vehicle group *p < 0.05; **p < 0.01; ***p < 0.001; **p < 0.0001 (ANOVA is followed by the Bonferroni’s post hoc test), *N* = 10

ADN-1184 at a dose of 0.625 mg/kg only produced a significant decrease in the number of buried marbles (ANOVA *F*(4,41) = 3.06, *p* < 0.05) during 30-min experimental period. Other doses did not reach significant level of the effect (Fig. 4a). Risperidone significantly decreased the number of buried marbles after administration at a dose of 0.01 mg/kg only (ANOVA *F*(3,34) = 6.563, *p* < 0.01) (Fig. 4b). Olanzapine (ANOVA *F*(3,30) = 31.873, *p* < 0.00001) and aripiprazole (ANOVA *F*(3,28) = 13.905, *p* < 0.00001) given at doses of 2.5 and 5 mg/kg significantly decreased the number of buried marbles (Fig. 4c, d).

## Discussion

ADN-1184, a novel monoaminergic ligand with antipsychotic and antidepressant activities (Kołaczkowski et al. 2014a), possesses a multitarget profile distinct from current atypical antipsychotic agents. Its dopamine D_2_ and D_3_ antagonist properties, together with its potent blockade of 5-HT_6_, 5-HT_7_ and 5-HT_2A_ receptors, and the absence of anticholinergic and antihistaminic effects, are consistent with a beneficial influence on psychosis, cognition, and mood impairments. Inasmuch as anxiety is a common and comorbid symptom of BPSD, we examined the potential action of ADN-1184 in common rodent models of anxiety to broaden its behavioral profile. We used different anxiety procedures based on a variety of anxiety stimuli. The EPM test is postulated to induce unconditioned fear due to heights and open spaces, which seems to be consistent with panic anxiety (Graeff et al. 1996). In this model, ADN-1184 increased all parameters measured; however, it only significantly increased the number of entries into open arms after administration at a dose of 0.3 mg/kg. Simultaneously, that dose of ADN-1184 increased parameters of exploratory activity indicating that this is likely accounted for the increased number of entries into the open arms.

Generalized anxiety disorder, a common, undertreated yet incapacitating disorder reveals a chronic prognosis and is frequently comorbid with other symptoms of BPSD (Liperoti et al. 2008). This anxiety state is probably linked to a bias in anticipating adverse circumstances, often without any obvious threat (Steimer 2011). There are few, if any, animal models sufficiently validated to discriminate among the various subtypes of anxiety disorders. In general, operant conflict procedures are pharmacologically isomorphic with the human state of generalized anxiety (Schlaepfer and Nemeroff 2012). Among these conditioned procedures, the Vogel test is of rather broad significance to clinical anxiety, particularly to generalized anxiety disorder (Millan and Brocco 2003). The anxiolytic-like activity of ADN-1184 is clearly visible in the Vogel conflict drinking test in rats, a “conditional” anxiety-like paradigm. ADN-1184 dose-dependently and significantly increased the number of shocks accepted and the number of licks. Moreover, it presented specific anti-conflict action, since ADN-1184 had an effect on neither the pain threshold nor unpunished water consumption. ADN-1184 also had specific anxiolytic-like activity in the FPT and only a negligible effect in the MBT visible at a specific mid-range dose. Thus, it appears that ADN-1184 clearly exerts the anxiolytic-like effect in tests incorporating punishing stimuli; however, it produces little or no effect in models based on innate anxiety in the EPM or anxiety/compulsive-like behavior in the MBT.

In the present study, we compared the action of ADN-1184 with the effects produced by three SGAs—olanzapine, aripiprazole, and risperidone—since the recent meta-analysis had revealed that all these drugs produced a small but significant improving effect on behavioral symptoms of dementia in elderly patients (Maher et al. 2011). It is noteworthy that, quantitatively, ADN-1184 was more efficacious in the FPT in mice than olanzapine and aripiprazole studied under the same conditions. However, in the EPM or MBT, it had no appreciable effect but risperidone does or olanzapine and aripiprazole do, respectively. In the rat Vogel model, ADN-1184 showed superiority over aripiprazole when one considers the active dose of both compounds. However, when the magnitude of change is taken into account, the studied compound did not present better parameters than the reference antipsychotic drugs. Furthermore, anxiolytic-like activity of ADN-1184 in both rats and mice was 10 to 30-fold weaker than that of risperidone acting in low doses; however, risperidone has greater potency to induce adverse effects, e.g., catalepsy and sedation, than ADN-1184 (Kołaczkowski et al. 2014a, b). These data deliver indications of accentuated anxiolytic-like activity of ADN-1184, especially in models employing punished stimuli; however, drawing definite conclusions about its clinical potency is premature. Generally, animal tests and in this case, animal models of anxiety can certainly be useful to find out more about a pharmacological profile of newly developed compounds and biological bases of anxiety disorders. These models certainly provide a lot of information on the brain and behavioral mechanisms which could be involved in the etiology and physiopathology of anxiety disorders, but are usually not satisfactory when confronted directly with clinical syndromes (Steimer 2011).

The neurobiological mechanisms that mediate the anxiolytic-like action of ADN-1184 are not clear. Since ADN-1184, similar to most SGAs, has a complex multiple-receptor binding profile, it is difficult to pinpoint which receptor action or actions account for its anxiolytic-like activity. Nevertheless, given the in vitro pharmacological profile of ADN-1184, it seems that 5-HT_6_ and/or 5-HT_7_ receptor antagonism is most probably involved in the anxiolytic-like effects of this compound. Indeed, it was previously shown that both the selective 5-HT_6_ and the selective 5-HT_7_ receptor antagonists displayed pronounced anti-anxiety effects, in the same animal models as the ones used for characteristic of ADN-1184 (Wesołowska and Nikiforuk 2007; Wesołowska et al. 2006). Furthermore, the described effects of 5-HT_6_ receptor blockade were more pronounced and were suggested to be connected with indirect enhancement of GABA-ergic transmission (Wesołowska 2008). Future research should be directed at clarifying this issue.

In conclusion, the obtained findings reveal that ADN-1184 displays anxiolytic-like activity in animal models of anxiety which employed punished stimuli. In its unusual combination of some anxiolytic action with already proven antipsychotic and antidepressant properties, and lack of any disruptive impact on learning and memory processes and motor coordination, ADN-1184 displays a profile, that would be desired for a novel therapeutic for BPSD. Additionally, considering any further development of pharmacotherapeutics to treat BPSD, one should bear in mind that the use of antipsychotic drugs in BPSD is limited by boxed warnings issued by the US Food and Drug Administration. The warnings added to the package inserts of SGAs emphasize the risk of serious adverse effects that can be induced by these drugs in elderly patients (Schneider et al. 2006; Schulze et al. 2013). Hence, meeting the need for efficacious medications to treat BPSD would require a continuous discussion between preclinical and clinical researchers as well as regulatory bodies and development of guidelines indicating strategies for evaluation of novel compounds with safer therapeutic profiles (Kołaczkowski et al. 2014a).
